# Prevalence and cardiometabolic correlates of ketohexokinase gene variants among UK Biobank participants

**DOI:** 10.1371/journal.pone.0247683

**Published:** 2021-02-23

**Authors:** Joseph A. Johnston, David R. Nelson, Pallav Bhatnagar, Sarah E. Curtis, Yu Chen, James G. MacKrell

**Affiliations:** 1 Global Patient Outcomes and Real World Evidence, Eli Lilly and Company, Indianapolis, Indiana, United States of America; 2 Lilly Research Laboratories, Eli Lilly and Company, Indianapolis, Indiana, United States of America; Stellenbosch University Faculty of Medicine and Health Sciences, SOUTH AFRICA

## Abstract

Essential fructosuria (EF) is a benign, asymptomatic, autosomal recessive condition caused by loss-of-function variants in the ketohexokinase gene and characterized by intermittent appearance of fructose in the urine. Despite a basic understanding of the genetic and molecular basis of EF, relatively little is known about the long-term clinical consequences of ketohexokinase gene variants. We examined the frequency of ketohexokinase variants in the UK Biobank sample and compared the cardiometabolic profiles of groups of individuals with and without these variants alone or in combination. Study cohorts consisted of groups of participants defined based on the presence of one or more of the five ketohexokinase gene variants tested for in the Affymetrix assays used by the UK Biobank. The rs2304681:G>A (p.Val49Ile) variant was present on more than one-third (36.8%) of chromosomes; other variant alleles were rare (<1%). No participants with the compound heterozygous genotype present in subjects exhibiting the EF phenotype in the literature (Gly40Arg/Ala43Thr) were identified. The rs2304681:G>A (p.Val49Ile), rs41288797 (p.Val188Met), and rs114353144 (p.Val264Ile) variants were more common in white versus non-white participants. Otherwise, few statistically or clinically significant differences were observed after adjustment for multiple comparisons. These findings reinforce the current understanding of EF as a rare, benign, autosomal recessive condition.

## Introduction

Essential fructosuria (EF) (OMIM #229800) is an autosomal recessive condition caused by loss-of-function variants in the ketohexokinase (KHK) gene (HGNC:6315) and characterized by intermittent appearance of fructose in the urine [1]. Its prevalence has been estimated to be 1:130,000 [2]. However, given that EF is asymptomatic, presumed benign, and does not require treatment, it may be more prevalent than reported. In seminal work on EF in the 1960’s, Laron identified 50 published cases of EF and noted that 18 of those were of Jewish decent [3], and Froesch commented that the anomaly was “encountered almost exclusively in Jews.” [4] Not surprisingly, given the benign nature of the EF, limited additional population characterization of EF has been performed since that time.

Most of what is known clinically about EF stems from the study of a single family in which variant analysis was performed and affected individuals were found to be compound heterozygotes for two variants resulting in amino acid substitutions of Gly40Arg and Ala43Thr in highly conserved regions of the KHK protein [1, 5, 6]. In contrast, Val49Ile, another common variant in normal Europeans, does not produce fructosuria when present in homozygous state [1]. Two KHK protein isotypes result from alternative splicing of the KHK gene, one with a “central,” predominantly hepatic distribution (i.e., KHK-C) and a second that is more widely distributed (i.e., KHK-A) [7]. Gly40Arg has been shown to result in no KHK activity in either isotype, while Ala43Thr leads to reduced thermostability of both isotypes [7]. The importance of additional variants (e.g., Val188Met and Val264Ile) is unknown.

Despite a basic understanding of the genetic and molecular basis of EF, relatively little is known about the clinical consequences, if any, of carrying KHK gene variants. Given the high concentration of fructose in the typical Western diet, inhibiting the function of KHK and the normal processing of fructose is an intriguing potential target for drug development, and understanding the cardiometabolic characteristics of humans with KHK gene variants may provide insight into the viability of this potential therapeutic approach. Our goal was to examine the frequency of KHK variants in the UK Biobank sample, including those implicated in EF, and to compare the cardiometabolic profiles of groups of individuals with and without these variants alone or in combination.

## Materials and methods

### Data sources

We used data from the UK Biobank, which recruited more than 500,000 participants out of 9.2 million eligible adults between the ages of 40 and 70 years in the UK who were invited to participate (5.5% response rate) [8]. The study protocol is available online [9] and more details are published elsewhere [10]. During the baseline assessment, participants provided informed consent and completed a questionnaire and interview covering a broad range of sociodemographic characteristics, health-related risk factors and behaviors, and past medical history. Assessment centre staff conducted a variety of physical measurements and collected blood and urine samples that were subsequently used to generate biomarker and genotype information. Information on subsequent death, disease occurrence and other health-related information are collected during follow-up from a variety of sources and systems, including death and cancer registries and general practice and hospital activity records.

This research was conducted using the UK Biobank Resource under Application Number 43833 and is covered by the ethics approval for UK Biobank studies from the NHS National Research Ethics Service (16/NW/0274). Each person who agreed to join the UK Biobank project received a clear assurance that all of their personal information would be held in strict confidence with careful controls, and that identifiable information about them would not be available to anyone outside the UK Biobank.

### Study populations

Study cohorts consisted of groups of participants defined based on the presence of one or more of the five KHK gene variants tested for in the Affymetrix assays used by the UK Biobank: rs104893643:G>A (p.Gly40Arg), rs104893644:G>A (p.Ala43Thr), rs2304681:G>A (p.Val49Ile), rs41288797:G>A (p.Val188Met) and rs114353144:G>A (p.Val264Ile). We chose to focus on only directly genotyped, non-synonymous, exonic variants as we felt this would ensure the highest level of confidence in terms of both data quality and pathogenicity. Participants (n = 14,241 [2.8%]) with missing data for all five of these loci we excluded. The corresponding Affymetrix identifiers, reference sequences and allele frequencies are shown in **Table 1**. Genomic coordinates are in reference to NCBI build GRCh38.p12.

**Table 1:** Frequency of ketohexokinase variants in UK Biobank.

dbSNP rs ID	Affy ID	Gene Variant*	Protein Variant	Allele Frequency^†^
rs104893643	Affx-80216441	NC_000002.12:g.27092357G>A	p.Gly40Arg	248 (0.03%)
rs104893644	Affx-80216442	NC_000002.12:g.27092366G>A	p.Ala43Thr	50 (0.01%)
rs2304681	Affx-19853162	NC_000002.12:g.27092384G>A	p.Val49Ile	358,641 (36.78%)
rs41288797	Affx-19853206	NC_000002.12:g.27097647G>A	p.Val188Met	895 (0.09%)
rs114353144	Affx-36290456	NC_000002.12:g.27099556G>A	p.Val264Ile	1,914 (0.20%)

Affy ID = unique Affymetrix SNP identifier; Ala = alanine; Arg = arginine; dbSNP rs ID = SNP identifier from dbSNP; Gly = glycine; Ile = isoleucine; Met = methionine; Thr = threonine; UK = United Kingdom; Val = valine.

*Genomic coordinates are in reference to NCBI build GRCh38.p12.

^†^Denominators range from 482,957 to 487,956 participants and 965,914 to 975,912 alleles.

Given the low prevalence of the variants other than p.Val49Ile, we compared subgroups of participants with each rare variant to those without the corresponding variant as a reference group. Given the high prevalence of p.Val49Ile, we compared groups with no, one and two such variants. Finally, as an exploratory analysis, we compared participants with a p.Val49Ile variant in combination with other variants to those with no variants. This was done to determine, given the autosomal recessive character of EF, whether participants with compound heterozygosity for Val49Ile and other KHK variants might manifest higher rates of cardiometabolic complications. In doing so, we examined separately those variants with known (i.e., Gly40Arg or Ala43Thr) and unknown (i.e., Val188Met or Val264Ile) associations with EF in the literature.

### Variables and measures

The UK Biobank contains genotypes for 488,377 participants who were successfully genotyped, 49,950 using the Applied Biosystems UK Biobank Lung Exome Variant Evaluation (BiLEVE) Axiom Array by Affymetrix (now part of Thermo Fisher Scientific) and 438,427 using the closely related Applied Biosystems UK Biobank Axiom Array [11]. Pre-imputation quality control, phasing and imputation of the UK Biobank genetic data have been described elsewhere [11].

We requested and analyzed all variables potentially associated with the cardiometabolic status of participants. These included age, gender, race and ethnicity, and select baseline participant-reported cardiovascular, gastrointestinal, renal, and endocrine medical conditions. We also examined use of certain medications (i.e., cholesterol lowering agents, antihypertensives, insulin), age at myocardial infarction, stroke, and death, and primary and contributory causes of death. We analyzed physical exam and laboratory features related to cardiometabolic health, including body mass index, lipid biomarkers, HbA1c, serum metabolic panel, and liver function tests. For analysis of lipid biomarkers and HbA1c, we excluded participants taking cholesterol-lowering medications and those with diabetes, respectively, as determined by participant self-report on the baseline assessment questionnaire. Finally, abdominal subcutaneous adipose tissue volume, total trunk fat volume and visceral adipose tissue volume, as determined using magnetic resonance imaging, were examined for the subset of participants in the UK Biobank multi-modal imaging substudy [12]. The full list of variables used is shown in **Table 3**.

### Statistical analyses

Pairwise comparisons of characteristics were made between groups with the following variant genotypes and those with corresponding reference sequences: rs104893643:G>A (p.Gly40Arg); rs104893644:G>A (p.Ala43Thr); rs41288797:G>A (p.Val188Met); rs114353144:G>A (p.Val264Ile); [rs2304681:G>A];[rs2304681: = ] (i.e., heterozygous Val49Ile); [rs2304681:G>A];[rs2304681:G>A] (i.e., homozygous Val49Ile); all compound heterozygotes with at least one Gly40Arg or Ala43Thr variant; and all compound heterozygotes with any two variants other than Gly40Arg and Ala43Thr. Demographic comparisons were made using Chi-square tests for categorical variables, (i.e., sex and ethnicity) and Student’s t-tests for continuous variables (i.e., age). All other comparisons were made using separate age- and sex-adjusted logistic/linear regression models for each binary/continuous trait of interest. We were unable to adjust for ethnicity directly due to small sample sizes. Instead, we conducted a sensitivity analysis by repeating all analyses among white participants only and noted instances where results from the sensitivity analysis were discrepant from those from the full cohort in table footnotes.

All p-values for the regression coefficients corresponding to the genetic variant groups were adjusted using the FDR method of Benjamini and Hochberg [13], with the exception of exploratory p-values in the **S1 Table**, which are not adjusted for multiplicity. The p-values for the sensitivity analysis were adjusted for multiplicity separately. Continuous variables are summarized as mean (standard deviation). All statistical analyses were conducted using SAS 9.4 software (SAS Institute Inc., Cary, NC, USA).

## Results

The frequency of KHK missense variant alleles in the UK Biobank sample is shown in **Table 1**. The Val49Ile variant was observed with a 36.8% frequency, and more than half of participants (60.0%) had at least one such variant. Other variant alleles were rare (<1%) in the studied sample. The joint frequency of KHK missense variants is shown in **Table 2**. Approximately 13% of participants carried Val49Ile variants on both KHK alleles, resulting in exclusively variant KHK protein (i.e., p.Val49Ile). Other variants in combination occurred in <1% (Val49Ile plus other) and <0.01% (any two other) of participants. No participants with the compound heterozygous Gly40Arg/Ala43Thr genotype (i.e., the combination present in subjects exhibiting the EF phenotype in the literature) were identified. All variants were in Hardy-Weinberg equilibrium, as evidenced by non-significant deviations of observed from expected genotype distributions for each of the variants (data not shown).

**Table 2:** Frequency of ketohexokinase haplotype combinations in UK Biobank*.

	Gly40Arg	Ala43Thr	Val49Ile	Val188Met	Val264Ile
**Gly40Arg**	0	0	207 (0.04%)	0	1 (<0.01%)
**Ala43Thr**	‒	0	44 (0.01%)	0	1 (<0.01%)
**Val49Ile**	‒	‒	66,155 (13.57%)	878 (0.18%)	734 (0.15%)
**Val188Met**	‒	‒	‒	1 (<0.01%)	2 (<0.01%)
**Val264Ile**	‒	‒	‒	‒	0

Ala = alanine; Arg = arginine; Gly = glycine; Ile = isoleucine; Met = methionine; Thr = threonine; UK = United Kingdom; Val = valine.

*Denominators range from 481,611 to 487,943 participants.

The comparison of demographic characteristics and cardiometabolic risk factors and outcomes for UK Biobank participants with and without rare KHK variants is shown in **Table 3**. We observed statistical significance between Val188Met (false discovery rate [FDR] p<0.0001) and Val264Ile (FDR p<0.0001) and white ethnicity as compared to participants without those variants. No other statistically significant differences were observed between the cohorts with any of the individual rare variants and those without, nor were any differences observed in the sensitivity analyses including only participants of white ethnicity.

**Table 3:** Comparison of cardiometabolic profile of UK Biobank participants with and without rare ketohexokinase variants*.

Characteristic	No Rare Variants (N = 477,686)	Gly40Arg Variant (n = 248)	Ala43Thr Variant (n = 50)	Val188Met Variant (n = 894)	Val264Ile Variant (n = 1,914)
Age at recruitment, mean (SD)	56.5 (8.1)	56.7 (7.7)	57.6 (8.3)	56.1 (8.2)	56.3 (8.1)
Female sex, %	54.2	56.5	66.0	56.0	55.2
Ethnic background, %					
White	94.2	98.8	98.0	98.5†	97.7†
Black or black British	1.6	0	0	0.2	0.6
Asian, Asian British, or Chinese	2.3	0	0	0.2	0.5
Mixed	0.6	0.8	0	0.2	0.6
Other	0.9	0.4	2.0	0.5	0.4
Unknown	0.1	0	0	0	0
Medical conditions (Cardiovascular), %	39.4	38.3	44.0	38.9	38.3
Hypertension	27.9	26.2	32.0	25.6	26.5
Angina	4.7	3.6	2.0	4.9	4.7
Myocardial infarction	3.8	2.4	2.0	3.6	3.8
Heart failure/pulmonary edema	1.6	0.4	0	1.1	1.5
Heart arrhythmia	5.4	5.2	6.0	4.5	5.9
Heart valve problem/heart murmur	5.3	4.4	4.0	4.8	5.6
Cardiomyopathy	3.0	2.0	0	2.1	2.5
Pericardial problem	3.0	1.6	2.0	2.2	2.6
Cerebrovascular disease	6.3	3.2	8.0	5.7	6.4
Peripheral vascular disease	4.9	2.8	4.0	4.4	5.0
Venous thromboembolic disease	4.1	2.4	2.0	2.8	4.0
High cholesterol	15.0	12.5	16.0	13.6	14.8
Medical conditions (Gastrointestinal), %	19.8	21.0	18.0	18.1	18.9
Esophageal disorder	10.0	9.3	4.0	9.1	10.0
Stomach disorder	4.0	4.0	2.0	3.5	3.9
Bowel problem (any)	10.9	8.1	16.0	10.3	11.1
Irritable bowel syndrome	3.9	2.8	4.0	3.4	4.0
Malabsorption/celiac disease	3.6	1.6	4.0	2.8	4.1
Constipation	1.9	1.2	0	1.5	1.4
Liver/biliary/pancreas problem	7.4	7.7	6.0	7.7	7.3
Medical conditions (Renal), %	5.5	3.2	4.0	5.9	6.1
Renal/kidney failure (any)	1.8	0.8	2.0	1.6	2.2
Requiring dialysis	1.7	0.8	2.0	1.6	2.1
Other renal/kidney problem (any)	4.8	2.4	4.0	5.2	5.2
Diabetic nephropathy	1.1	0.8	0	1.7	1.4
Medical conditions (Endocrine/Diabetes), %	14.9	14.9	14.0	15.8	14.0
Diabetes (any)	8.2	5.7	4.0	8.2	7.0
Type 2 diabetes	3.1	0.8	2.0	2.8	3.1
Other endocrine condition‡	6.7	9.3	10.0	7.6	7.0
Medical conditions (Other), %					
Diabetic neuropathy/ulcers	3.2	1.2	4.0	2.6	3.9
Age at event, mean (SD)					
Myocardial infarction	58.2 (13.7)	60.9 (7.5)	28.8 (59.2)	58.0 (8.9)	59.0 (8.7)
Stroke	57.4 (15.9)	56.2 (3.2)	63.4 (11.0)	48.9 (32.1)	58.3 (8.9)
Medication use, %					
Cholesterol lowering medication	11.2	8.9	6.0	12.3	11.2
Blood pressure medication	11.9	10.1	12.0	11.2	11.7
Insulin	0.7	0.8	0	0.8	0.8
Mortality, %	4.0	2.4	4.0	3.5	4.1
Age at death, mean (SD)	67.3 (7.1)	69.4 (5.8)	69.1 (2.2)	68.3 (7.1)	66.3 (7.0)
Primary cause of death, %					
Diseases of the circulatory system	0.8	0.4	0	0.6	1.0
Diseases of the digestive system	0.2	0	0	0.2	0.1
Contributory cause of death, %					
Diseases of the circulatory system	1.1	0.8	0	1.2	1.1
Diseases of the digestive system	0.2	0	0	0.3	0.2
Morphometrics, mean (SD) §					
Body mass index, kg/m^2^	27.4 (4.8)	27.0 (4.6)	26.8 (5.9)	27.5 (4.7)	27.1 (4.9)
Abdominal subcutaneous adipose tissue volume, L	6.5 (3.2)	-	-	5.0 (1.1)	5.7 (2.5)
Total trunk fat volume, L	10.3 (4.6)	-	-	7.9 (2.6)	8.9 (3.2)
Visceral adipose tissue volume, L ‖	3.2 (2.3)	-	-	2.3 (2.1)	2.8 (1.9)
Biomarkers (Cardiovascular), mean (SD) ¶					
Cholesterol (serum), mmol/L	5.8 (1.1)	5.8 (1.2)	5.6 (0.9)	5.8 (1.1)	5.8 (1.1)
LDL (serum), mmol/L	3.6 (0.8)	3.6 (0.9)	3.5 (0.7)	3.6 (0.8)	3.6 (0.8)
HDL (serum), mmol/L	1.5 (0.4)	1.5 (0.4)	1.6 (0.3)	1.5 (0.4)	1.5 (0.4)
Triglyceride (serum), mmol/L	1.7 (1.0)	1.7 (1.1)	1.5 (0.7)	1.7 (1.0)	1.7 (1.0)
Apolipoprotein A (serum), g/L	1.6 (0.3)	1.5 (0.3)	1.6 (0.2)	1.6 (0.3)	1.6 (0.3)
Apolipoprotein B (serum), g/L	1.1 (0.2)	1.0 (0.2)	1.0 (0.2)	1.0 (0.2)	1.0 (0.2)
C-reactive protein (serum), mg/L	2.6 (4.4)	2.9 (5.9)	1.9 (2.0)	2.6 (4.4)	2.4 (4.3)
Lipoprotein (a) (serum), nmol/L	44.3 (48.9)	49.6 (52.1)	41.3 (47.4)	42.8 (47.2)	44.0 (49.4)
Biomarkers (Diabetes), mean (SD) #					
HbA1c (RBC), mmol/mol	36.0 (6.5)	35.5 (5.0)	35.7 (4.2)	35.9 (7.0)	35.6 (5.9)
Glucose (serum), mmol/L	5.1 (1.2)	5.1 (1.0)	5.1 (1.2)	5.2 (1.3)	5.1 (1.1)
Biomarkers (Renal), mean (SD)					
Cystatin C (serum), mg/L	0.9 (0.2)	0.9 (0.2)	0.9 (0.1)	0.9 (0.1)	0.9 (0.2)
Creatinine (serum), *μ*mol/L	72.3 (18.7)	72.4 (15.7)	68.5 (10.9)	71.1 (14.4)	72.5 (18.6)
Total protein (serum), g/L	72.5 (4.1)	72.2 (4.0)	71.7 (3.6)	72.5 (4.0)	72.4 (4.1)
Urea (serum), mmol/L	5.4 (1.4)	5.5 (1.4)	5.2 (1.2)	5.4 (1.3)	5.4 (1.5)
Phosphate (serum), mmol/L	1.2 (0.2)	1.2 (0.2)	1.2 (0.1)	1.2 (0.2)	1.2 (0.2)
Urate (serum), *μ*mol/L	309.2 (80.4)	303.3 (71.4)	294.7 (67.9)	309.6 (84.2)	306.1 (81.4)
Biomarkers (Liver), mean (SD)					
Albumin (serum), g/L	45.2 (2.6)	45.1 (2.6)	44.5 (2.8)	45.2 (2.5)	45.2 (2.7)
Direct bilirubin (serum), *μ*mol/L	1.8 (0.9)	2.0 (0.9)	1.9 (0.8)	1.8 (0.8)	1.8 (0.8)
Gamma glutamyltransferase (serum), U/L	37.4 (42.2)	36.6 (42.6)	35.8 (32.2)	39.2 (52.3)	35.5 (34.3)
Alanine aminotransferase (serum), U/L	23.6 (14.2)	22.7 (11.6)	25.2 (12.7)	23.7 (12.8)	23.5 (16.3)
Aspartate aminotransferase (serum), U/L	26.2 (10.7)	25.8 (8.3)	27.4 (6.7)	26.3 (8.7)	26.3 (12.9)

Ala = alanine; Arg = arginine; Gly = glycine; HbA1c = hemoglobin A1c; HDL = high-density lipoprotein; Ile = isoleucine; LDL = low-density lipoprotein; Met = methionine; SD = standard deviation; Thr = threonine; Val = valine; RBC = red blood cell.

*For descriptive purposes, the “no rare variants” group includes only participants with reference sequences at all four loci. However, for statistical comparison between groups with and without individual variants, only those without the variant of interest were excluded from the reference group.

†*P* < 0.05 for comparison between participants with and without the corresponding KHK variant using false discovery rate adjustment.

‡Other endocrine conditions include those in *ICD-10* category E34, including “endocrine disorder, unspecified” (E34.9) and “carcinoid syndrome” (E34.0).

§Adipose tissue volumes were determined using abdominal magnetic resonance imaging and were only available for a small subset of participants (n = 5,994 [1.2%] overall, ranging from 0 [0%] to 20 [1.0%] within the four rare variant groups).

‖Visceral adipose tissue volume is the volume of adipose tissue within the abdominal cavity, excluding adipose tissue outside the abdominal skeletal muscles and adipose tissue and lipids within and posterior of the spine and posterior of the back muscles.

¶Analysis of lipid biomarkers excludes participants taking cholesterol-lowering medications.

#Analysis of diabetes biomarkers (i.e., HbA1c and glucose) excludes participants diagnosed with type 2 diabetes.

The comparison of demographic characteristics and cardiometabolic risk factors and outcomes for UK Biobank participants with and without Val49Ile KHK variants is shown in **Table 4**. Overall, few statistically significant differences were observed after adjustment for multiple comparisons. Participants with single Val49Ile variants were more frequently of white ethnicity (94.7% vs. 93.4%; FDR p<0.001) than those with no Val49Ile variants. They also had lower total trunk fat volume (10.1 ± 4.6L vs. 10.5 ± 4.6L; FDR p = 0.01) and visceral adipose tissue volume (3.1 ± 2.2L vs. 3.4 ± 2.3L; FDR p<0.001). These differences were also statistically significant among only participants of white ethnic background. Those with double Val49Ile variants were also more frequently of white ethnicity (95.3% vs. 93.4%; FDR p<0.001), less frequently diagnosed with hypertension (27.4% vs. 28.1%; FDR p = 0.001), more frequently diagnosed with endocrine conditions other than diabetes (7.0% vs. 6.6%; FDR p = 0.02), and had lower hemoglobin A1c (HbA1c) values (35.9 ± 6.6 mmol/mol vs. 36.0 ± 6.5 mmol/mol; FDR p = 0.003) than those with no Val49Ile variants; none of these differences were statistically significant among only participants of white ethnic background.

**Table 4:** Comparison of cardiometabolic profile of UK Biobank participants with and without Val49Ile ketohexokinase variants*.

Characteristic	No Val49Ile Variant (n = 195,103)	Single Val49Ile Variant (n = 226,319)	Double Val49Ile Variant (n = 66,155)
Age at recruitment, mean (SD)	56.5 (8.1)	56.5 (8.1)	56.6 (8.1)
Female sex, %	54.2	54.2	54.3
Ethnic background, %			
White	93.4	94.7†	95.3†
Black or black British	1.8	1.5	1.2
Asian, Asian British or Chinese	2.7	2.0	1.6
Mixed	0.6	0.6	0.5
Other	1.0	0.9	0.8
Unknown	0.1	0.1	0.1
Medical conditions (Cardiovascular), %	39.5	39.3	39.2
Hypertension	28.1	27.8	27.4†
Angina	4.7	4.7	4.6
Myocardial infarction	3.9	3.8	3.8
Heart failure/pulmonary edema	1.6	1.6	1.6
Heart arrhythmia	5.3	5.4	5.4
Heart valve problem/heart murmur	5.3	5.3	5.4
Cardiomyopathy	3.0	3.0	3.1
Pericardial problem	3.0	3.0	3.1
Cerebrovascular disease	6.2	6.2	6.4
Peripheral vascular disease	4.8	4.9	5.0
Venous thromboembolic disease	4.0	4.1	4.1
High cholesterol	15.0	14.9	15.0
Medical conditions (Gastrointestinal), %	19.7	19.8	20.2
Esophageal disorder	10.0	10.0	10.2
Stomach disorder	4.0	4.0	4.0
Bowel problem (any)	10.8	10.9	11.1
Irritable bowel syndrome	3.9	3.9	4.0
Malabsorption/celiac disease	3.5	3.6	3.6
Constipation	1.8	1.8	1.9
Liver/biliary/pancreas problem	7.3	7.4	7.5
Medical conditions (Renal), %	5.5	5.5	5.6
Renal/kidney failure (any)	1.8	1.8	1.8
Requiring dialysis	1.7	1.7	1.7
Other renal/kidney problem (any)	4.8	4.8	4.9
Diabetic nephropathy	1.1	1.1	1.2
Medical conditions (Endocrine/Diabetes), %	14.9	14.9	15.1
Diabetes (any)	8.4	8.2	8.1
Type 2 diabetes	3.0	3.0	3.1
Other endocrine condition §	6.6	6.7	7.0†
Medical conditions (Other), %			
Diabetic neuropathy/ulcers	3.2	3.2	3.3
Age at event, mean (SD)			
Myocardial infarction	57.9 (14.6)	58.4 (13.0)	58.1 (13.2)
Stroke	57.4 (15.8)	57.5 (15.9)	56.8 (16.6)
Medication use, %			
Cholesterol lowering medication	11.3	11.2	11.1
Blood pressure medication	11.9	11.8	11.7
Insulin	0.7	0.7	0.6
Mortality, %	4.0	4.0	4.0
Age at death, mean (SD)	67.3 (7.1)	67.3 (7.0)	67.2 (7.2)
Primary cause of death, %			
Diseases of the circulatory system	0.9	1.1	0.8
Diseases of the digestive system	0.2	0.2	0.2
Contributory cause of death, %			
Diseases of the circulatory system	1.1	1.1	1.1
Diseases of the digestive system	0.3	0.2	0.3
Morphometrics, mean (SD) ‖			
Body mass index, kg/m^2^	27.4 (4.8)	27.4 (4.8)	27.4 (4.8)
Abdominal subcutaneous adipose tissue volume, L	6.6 (3.3)	6.5 (3.2)	6.5 (3.1)
Total trunk fat volume, L	10.5 (4.6)	10.1 (4.6)†‡	10.1 (4.3)
Visceral adipose tissue volume, L ¶	3.4 (2.3)	3.1 (2.2)†‡	3.1 (2.2)
Biomarkers (Cardiovascular), mean (SD) #			
Cholesterol (serum), mmol/L	5.8 (1.1)	5.8 (1.1)	5.9 (1.1)
LDL (serum), mmol/L	3.6 (0.8)	3.6 (0.8)	3.7 (0.8)
HDL (serum), mmol/L	1.5 (0.4)	1.5 (0.4)	1.5 (0.4)
Triglyceride (serum), mmol/L	1.7 (1.0)	1.7 (1.0)	1.7 (1.0)
Apolipoprotein A (serum), g/L	1.6 (0.3)	1.6 (0.3)	1.6 (0.3)
Apolipoprotein B (serum), g/L	1.0 (0.2)	1.1 (0.2)	1.1 (0.2)
C-reactive protein (serum), mg/L	2.6 (4.4)	2.6 (4.3)	2.6 (4.3)
Lipoprotein (a) (serum), nmol/L	44.5 (48.8)	44.2 (48.9)	44.3 (49.9)
Biomarkers (Diabetes), mean (SD) **			
HbA1c (RBC), mmol/mol	36.0 (6.5)	36.0 (6.4)	35.9 (6.6)†
Glucose (serum), mmol/L	5.1 (1.2)	5.1 (1.2)	5.1 (1.1)
Biomarkers (Renal), mean (SD)			
Cystatin C (serum), mg/L	0.9 (0.2)	0.9 (0.2)	0.9 (0.2)
Creatinine (serum), *μ*mol/L	72.2 (17.9)	72.4 (19.0)	72.4 (19.5)
Total protein (serum), g/L	72.5 (4.1)	72.5 (4.1)	72.5 (4.1)‡
Urea (serum), mmol/L	5.4 (1.4)	5.4 (1.4)	5.4 (1.4)
Phosphate (serum), mmol/L	1.2 (0.2)	1.2 (0.2)	1.2 (0.2)
Urate (serum), *μ*mol/L	309.6 (80.5)	309.0 (80.3)	309.2 (80.5)
Biomarkers (Liver), mean (SD)			
Albumin (serum), g/L	45.2 (2.6)	45.2 (2.6)	45.2 (2.6)
Direct bilirubin (serum), *μ*mol/L	1.8 (0.9)	1.8 (0.8)	1.8 (0.8)
Gamma glutamyltransferase (serum), U/L	37.4 (42.5)	37.3 (41.7)	37.5 (42.3)
Alanine aminotransferase (serum), U/L	23.6 (14.2)	23.5 (14.2)	23.5 (14.3)
Aspartate aminotransferase (serum), U/L	26.2 (10.7)	26.2 (10.8)	26.2 (10.2)

Abbreviations are explained in the first footnote to **Table 3.**

*Val49Ile genotypes were unavailable for 675 (0.14%) individuals.

†*P* < 0.05 for unadjusted (age, sex, ethnicity) or age- and sex-adjusted (all others) comparison to participants with no Val49Ile variants (GG) using false discovery rate adjustment.

‡*P* < 0.05 in sensitivity analysis including only participants of white ethnic background.

§Other endocrine conditions include those in *ICD-10* category E34, including “endocrine disorder, unspecified” (E34.9) and “carcinoid syndrome” (E34.0).

‖Adipose tissue volumes were determined using abdominal magnetic resonance imaging and were only available for a small subset of participants (n = 5,994 [1.2%] overall, ranging from 822 [1.3%] to 2,667 [1.3%] within the three variant subgroups).

¶Visceral adipose tissue volume is the volume of adipose tissue within the abdominal cavity, excluding adipose tissue outside the abdominal skeletal muscles and adipose tissue and lipids within and posterior of the spine and posterior of the back muscles.

#Analysis of lipid biomarkers excludes participants taking cholesterol-lowering medications.

**Analysis of diabetes biomarkers (i.e., HbA1c and glucose) excludes participants diagnosed with type 2 diabetes.

Given the autosomal recessive inheritance nature of EF, we were also interested in participants with multiple KHK variants. Differences between participants with two KHK variants and those with none are shown in **S1 Table**. Note that, while a number of statistically significant differences were observed, only one, an increased direct bilirubin in the group with two KHK variants including Gly40Arg or Ala43Thr (1.9 [0.9] μmol/L vs. 1.8 [0.9] μmol/L), was significant in both age- and sex-adjusted analyses and in the sensitivity analysis among only white participants. These findings should be considered exploratory only as these p-values were not adjusted for multiple comparisons and the variation was within the normal range.

## Discussion

Our failure to identify even a single individual with the Gly40Arg/Ala43Thr genotype among UK Biobank participants suggests either that the prevalence of EF is much lower than previously estimated or that there may be important differences in prevalence by race and ethnicity. Based on the carrier frequency of these two variants in the UK Biobank, and the fact that they are in Hardy-Weinberg equilibrium, we can estimate the prevalence of EF to be approximately 0.37:1,000,000, considerably lower than published estimates. To contextualize the variant allele frequencies observed in the UK Biobank sample, we queried these five KHK variants in the Genome Aggregation Database (gnomAD https://gnomad.broadinstitute.org/), a public resource seeking to aggregate and harmonize exome and genome sequencing data from a variety of large-scale sequencing projects [14]. This resource (gnomAD v2) contains data from 125,748 exomes and 15,708 whole genomes, and **S2 Table** shows the variant allele frequency distributions for different global populations. In general, allele frequencies in the UK Biobank sample were similar to those in the European non-Finnish population from gnomAD. However, it should be noted that, while we found that UK Biobank participants with Val49Ile, Val188Met, and Val264Ile variants were more frequently of white ethnicity than those with no variants, the overwhelming majority of UK Biobank participants (94.1%) are in fact white, and these variants are equally and in some cases more prevalent in other ethnic groups, in particular Latinos (**S2 Table**). Further insights into the geographic and ethnic distribution of KHK variants could be obtained by replicating this study in other large biobank cohorts from China or Scandinavia [15–17].

Previous literature has suggested that compound heterozygosity is required for the EF phenotype based on serum and urinary fructose analysis. Since the UK Biobank biomarker panels did not include serum or urinary fructose, we could not confirm this; however, our findings do suggest that neither individual KHK variants nor compound heterozygosity for the variant combinations we identified appear to confer a decreased risk of fructose-mediated adverse cardiometabolic events. In particular, the fact that the few statistically significant differences observed between individuals with a single Val49Ile variant and those with none were not also observed in those with two Val49Ile variants, and that the differences observed in those with two variants were not replicated among white participants only suggests that the Val49Ile KHK protein is likely a normally functioning variant. Alternatively, it is possible that the Val49Ile variant does indeed have cardiometabolic consequences but that they are of small magnitude and that we are thus unable to be demonstrate statistical significance despite the large numbers of participants with this common variant.

To date, it remains unclear from the published literature whether the variants studied, other than Gly40Arg and Ala43Thr, are functional. Unfortunately, using the UK Biobank data we were unable to provide any further evidence regarding cardiometabolic outcomes associated with the Gly40Arg/Ala43Thr genotype, or to characterize sufficient participants with multiple non-Val49Ile variants, to derive meaningful conclusions about the clinical significance of the Val188Met and Val264Ile variants. While the decreased prevalence of diabetes and slightly lower BMIs in participants with two KHK variants including Gly40Arg or Ala43Thr is intriguing, it should be noted these findings were not replicated in the sensitivity analysis including only white participants. In this case, the small numbers of participants with rare variants in combination limit our power to establish meaningful differences.

Preclinical data in mice have shown that full genetic knockout of KHK (KHK-KO) provides cardiometabolic protection/benefits under various metabolic stressors [18]. While we hoped to be able to determine whether the EF genotype (akin to preclinical KHK-KO) conferred any clinical benefit in humans, we were barred from doing so by an absence of identifiable EF subjects in the UK Biobank. On the other hand, this dataset did allow us to identify participants with single variants (e.g., Gly40Arg or Ala43Thr). In these individuals, partial loss in protein function appeared neither to confer any clinical benefit, nor to result in any negative, detrimental phenotypes.

Interestingly, participants homozygous for the relatively common Val49Ile were marginally less frequently diagnosed with hypertension. Compelling evidence from basic science, population studies, and clinical trials implicate fructose as playing a major role in the development of hypertension, among an array of additional metabolic complications [19–29]. In line with these findings, removing dietary fructose has resulted in reversal of adverse metabolic effects, including hypertension. The mechanism connecting fructose to hypertension is unclear and may be an indirect product of worsened metabolic phenotype. However, there is emerging evidence that fructose metabolism in specific cell types (e.g., kidney/proximal tubule and vascular endothelium) directly promote pathways involved in increased blood pressure, including renal NHE3 upregulation [30–34]. While the decreased incidence of hypertension in participants with two Val49Ile variants in this dataset is encouraging, the lack of additional clinical benefits (e.g., decreased glucose, insulin, and triglycerides, as seen in fructose restriction and/or clinical KHK inhibition), as well as the fact that this association was not statistically significant among only white participants, complicates interpretation and application of the data. In addition, the Val49Ile variant has previously been suggested to have no functional importance [1, 7]. Nevertheless, these data do confirm that variants within KHK do not appear to result in detrimental phenotypes and suggest additional biological exploration into this finding (e.g., preclinical Val49Ile characterization) is warranted.

Recent clinical data also suggest that submaximal pharmacological KHK inhibition may provide clinically meaningful benefits. In a phase 2a study (NCT03256526), administration of Pfizer’s KHK inhibitor compound (PF06835919) at doses of 75 mg or 300 mg for 6 weeks was evaluated in subjects with non-alcoholic fatty liver disease (NAFLD) [35]. A significant reduction in whole liver fat, as measured by magnetic resonance imaging proton density fat fraction in subjects with NAFLD, was observed at the 300 mg per day dose. At both the 75 mg and 300 mg doses, significant improvements in a marker of insulin sensitivity (i.e., Homeostatic Model Assessment of Insulin Resistance) were observed. Noteworthy was the dose-response in efficacy observed from 75 mg to 300 mg on high-sensitivity C-reactive protein and adiponectin, markers of inflammation and insulin sensitivity, respectively, suggesting submaximal pharmacological KHK inhibition may result in clinical benefit. However, without knowing the target engagement profile of PF06835919 and lacking any EF subject data to characterize, it remains to be determined if pharmacological KHK inhibition is comparable to genetic (partial or full) loss of KHK function.

While no participants with EF were identified in this dataset, other investigators [36] have derived relevant insights from a model simulating fructose fate in the liver compartment of normal and EF individuals. Their model predicted that in EF (vs. normal/baseline), increased urinary fructose is a result of decreased reabsorption by the kidneys; i.e., the rate of fructose reabsorption in normal subjects would be higher than individuals with EF even when exposed to similar fructose levels in the kidney. The model suggests that, in the liver of EF subjects, non-KHK metabolism increases while peripheral fructose metabolism slows. The authors conclude that EF adaptations may be a compensatory mechanism to avoid malabsorption of fructose and maximize non-KHK metabolism. Whether these predicted adaptations would mask any potential clinical benefit of genetic KHK loss-of-function (partial or full) relative to those observed with clinical pharmacological KHK inhibition will be revealed upon more robust characterization of EF subjects. Nonetheless, the clinical benefit observed with pharmacological inhibition suggests that KHK may be a promising therapeutic target for cardiometabolic diseases including NAFLD and type 2 diabetes mellitus.

Several limitations deserve mention. The UK Biobank cohort, while large and well characterized, includes only UK citizens between the ages of 40 and 70, is not a representative sample of all UK citizens and is known to be biased towards healthier individuals [37]. It is unknown how well our findings regarding the prevalence of KHK variants in this sample would translate to other geographically diverse populations. Another limitation pertains to available data elements. Despite the richness of the UK Biobank dataset, several key biomarkers that could have provided meaningful insights into KHK functioning, including urinary fructose excretion, serum fructose after a fructose bolus, and liver fat, were unavailable [3, 38, 39].

Despite these limitations, we believe our findings, based on a sample of nearly half a million participants, add to what is known about the prevalence and the clinical consequences of KHK variants singly and in combination. These findings reinforce the current understanding of EF as a rare, benign, autosomal recessive condition. While we observed no apparent clinical benefit in participants with compound heterozygous KHK variants, we cannot on that basis alone exclude the possibility that certain variants might produce the EF phenotype and result in meaningful cardiometabolic benefits.

## Supporting information

S1 Table Comparison of cardiometabolic profile of UK Biobank participants with multiple ketohexokinase variants. (DOCX)

S2 Table Rare allele frequencies of ketohexokinase variants in gnomAD. (DOCX)
