# Supplementary material for: Prevalence and cardiometabolic correlates of ketohexokinase gene variants among UK Biobank participants
Source: PLoS One. 2021 Feb 23;16(2):e0247683. doi: 10.1371/journal.pone.0247683 (PMC7901775; doi:10.1371/journal.pone.0247683)
Supplement: S1 Table — (DOCX) [file pone.0247683.s001.docx]

**S1 Table**. **Comparison of cardiometabolic profile of UK Biobank participants with multiple ketohexokinase variants**

| **Characteristic** | **No KHK**  **Variants (n=190,916**) | **Two KHK Variants Including Gly40Arg or Ala43Thr (n=251**) | **Two KHK Variants Excluding Gly40Arg or Ala43Thr (n=1,608)** |
| --- | --- | --- | --- |
| Age at recruitment, mean (SD) | 56.5 (8.1) | 56.7 (7.8) | 56.1 (8.1)* |
| Female sex, % | 54.2 | 57.0 | 55.6 |
| Ethnic background, %  White  Black or black British  Asian, Asian British or Chinese  Mixed  Other  Unknown | 93.4  1.8  2.7  0.6  1.0  0.1 | 98.4*  0  0  0.8  0.8  0 | 98.5*  0.3  0.2  0.3  0.4  0 |
| Medical conditions (Cardiovascular), %  Hypertension  Angina  Myocardial infarction  Heart failure/pulmonary edema  Heart arrhythmia  Heart valve problem/heart murmur  Cardiomyopathy  Pericardial problem  Cerebrovascular disease  Peripheral vascular disease  Venous thromboembolic disease  High cholesterol | 39.5  28.2  4.7  3.9  1.6  5.3  5.3  3.0  3.0  6.2  4.8  4.0  15.0 | 38.3  26.7  3.2  2.8  0.4  4.4  4.8  1.6  2.0  4.8  3.6  2.4  13.6 | 38.5  25.9  4.9  3.8  1.4  5.5  5.7  2.4  2.6  6.6  5.0  3.0†  14.6 |
| Medical conditions (Gastrointestinal), %  Esophageal disorder  Stomach disorder  Bowel problem (any)  Irritable bowel syndrome  Malabsorption/celiac disease  Constipation  Liver/biliary/pancreas problem | 19.7  10.0  4.0  10.8  3.9  3.5  1.8  7.3 | 22.3  8.8  4.0  10.4  2.8  2.4  1.2  8.0 | 18.9  9.5  3.4  11.4  4.0  3.8  1.4  7.8 |
| Medical conditions (Renal), %  Renal/kidney failure (any)  Requiring dialysis  Other renal/kidney problem (any)  Diabetic nephropathy | 5.5  1.8  1.7  4.8  1.1 | 3.6  0.8  0.8  3.2  0.8 | 6.3  1.9  1.8  5.6  1.6 |
| Medical conditions (Endocrine/Diabetes), %  Diabetes (any)  Type 2 diabetes  Other endocrine condition ‡ | 15.0  8.4  3.0  6.6 | 13.6  4.8*  0.8  8.8 | 15.0  7.7  3.2  7.3 |
| Medical conditions (Other), %  Diabetic neuropathy/ulcers | 3.2 | 2.0 | 3.5 |
| Age at event, mean (SD)  Myocardial infarction  Stroke | 57.9 (14.6)  57.4 (15.8) | 51.3 (32.5)  59.8 (7.8) | 59.3 (8.8)  53.3 (24.2) |
| Medication use, %  Cholesterol lowering medication  Blood pressure medication  Insulin | 11.4  12.0  0.7 | 8.8  10.4  0.4 | 11.8  11.9  0.8 |
| Mortality, % | 4.0 | 2.8 | 3.9 |
| Age at death, mean (SD) | 67.3 (7.1) | 69.3 (5.4) | 67.4 (6.9) |
| Primary cause of death, %  Diseases of the circulatory system  Diseases of the digestive system | 0.9  0.2 | 0.4  0 | 0.9  0.2 |
| Contributory cause of death, %  Diseases of the circulatory system  Diseases of the digestive system | 1.1  0.3 | 0.8  0 | 1.2  0.2 |
| Morphometrics, mean (SD) §  Body mass index, kg/m^2^  Abdominal subcutaneous adipose tissue volume, L  Total trunk fat volume, L  Visceral adipose tissue volume, L‖ | 27.4 (4.8)  6.6 (3.3)  10.6 (4.6)  3.4 (2.3) | 26.8 (4.9)*  -  -  - | 27.3 (4.8)  5.6 (2.4)†  8.6 (3.1)†  2.5 (1.9) |
| Biomarkers (Cardiovascular), mean (SD) ¶  Cholesterol (serum), mmol/L  LDL (serum), mmol/L  HDL (serum), mmol/L  Triglyceride (serum), mmol/L  Apolipoprotein A (serum), g/L  Apolipoprotein B (serum), g/L  C-reactive protein (serum), mg/L  Lipoprotein (a) (serum), nmol/L | 5.8 (1.1)  3.6 (0.8)  1.5 (0.4)  1.7 (1.0)  1.6 (0.3)  1.0 (0.2)  2.6 (4.4)  44.4 (48.8) | 5.7 (1.1)  3.6 (0.8)  1.5 (0.4)  1.7 (1.0)  1.6 (0.3)  1.0 (0.2)  2.8 (5.9)  50.4 (52.0) | 5.8 (1.1)  3.7 (0.8)  1.5 (0.4)  1.7 (1.0)  1.6 (0.3)  1.0 (0.2)  2.5 (4.4)  42.4 (47.2) |
| Biomarkers (Diabetes), mean (SD) #  HbA1c (RBC), mmol/mol  Glucose (serum), mmol/L | 36.2 (6.8)  5.1 (1.3) | 35.3 (4.8)  5.1 (1.1) | 35.7 (6.4)  5.1 (1.2) |
| Biomarkers (Renal), mean (SD)  Cystatin C (serum), mg/L  Creatinine (serum), *μ*mol/L  Total protein (serum), g/L  Urea (serum), mmol/L  Phosphate (serum), mmol/L  Urate (serum), *μ*mol/L | 0.9 (0.2)  72.2 (17.9)  72.5 (4.1)  5.4 (1.4)  1.2 (0.2)  309.6 (80.6) | 0.9 (0.2)  72.0 (14.9)  72.0 (4.0)  5.4 (1.4)  1.2 (0.2)  301.6 (69.5) | 0.9 (0.2)  72.0 (19.0)  72.5 (4.0)  5.4 (1.5)  1.2 (0.2)  307.1 (84.4) |
| Biomarkers (Liver), mean (SD)  Albumin (serum), g/L  Direct bilirubin (serum), *μ*mol/L  Gamma glutamyltransferase (serum), U/L  Alanine aminotransferase (serum), U/L  Aspartate aminotransferase (serum), U/L | 45.2 (2.6)  1.8 (0.9)  37.4 (42.6)  23.5 (14.1)  26.2 (10.7) | 45.0 (2.7)  1.9 (0.9)*†  37.4 (43.2)  23.3 (11.8)  26.2 (8.4) | 45.3 (2.6)  1.8 (0.8)  36.7 (43.7)  23.3 (12.8)  26.0 (8.3) |

Abbreviations are explained in the first footnote to Table 3.

NOTE: Val49Ile genotypes were unavailable for 675 (0.14%) individuals. There were also 5 individuals with multiple KHK variants who were included in multiple categories in Table 2 but in only one here, accounting for the apparent difference in sample sizes.

^*^*P* < 0.05 for age- and sex-adjusted comparison to participants with no Val49Ile variants (GG); due to exploratory nature, no p-value adjustment.

†*P* < 0.05 in sensitivity analysis including only participants of white ethnic background.

‡Other endocrine conditions include those in *ICD-10* category E34, including “endocrine disorder, unspecified” (E34.9) and “carcinoid syndrome” (E34.0).

§Adipose tissue volumes were determined using abdominal magnetic resonance imaging and were only available for a subset of participants (n=5,994 [1.2%] overall, ranging from 0 [0%] to 2,262 [1.2%] within the three variant subgroups).

‖Visceral adipose tissue volume is the volume of adipose tissue within the abdominal cavity, excluding adipose tissue outside the abdominal skeletal muscles and adipose tissue and lipids within and posterior of the spine and posterior of the back muscles.

¶Analysis of lipid biomarkers excludes participants taking cholesterol-lowering medications.

#Analysis of diabetes biomarkers (i.e., HbA1c and glucose) excludes participants diagnosed with type 2 diabetes.
