# Supplementary material for: Prevalence and cardiometabolic correlates of ketohexokinase gene variants among UK Biobank participants
Source: PLoS One. 2021 Feb 23;16(2):e0247683. doi: 10.1371/journal.pone.0247683 (PMC7901775; doi:10.1371/journal.pone.0247683)
Supplement: S2 Table — (DOCX) [file pone.0247683.s002.docx]

**S2 Table. Rare allele frequencies of** **ketohexokinase variants in gnomAD**

| **Population** | **rs104893643** | **rs104893644** | **rs2304681** | **rs41288797** | **rs114353144** |
| --- | --- | --- | --- | --- | --- |
|  | **p.Gly40Arg** | **p.Ala43Thr** | **p.Val49Ile** | **p.Val188Met** | **p.Val264Ile** |
| African | 0.004% | 0% | 32.0% | 0.028% | 0.044% |
| Ashkenazi Jewish | 0% | 0% | 38.8% | 0.154% | 0% |
| East Asian | 0.020% | 0% | 23.5% | 0% | 0.005% |
| European (Finnish) | 0% | 0.004% | 33.5% | 0.040% | 0% |
| **European (non-Finnish)** | **0.022%** | **0.013%** | **37.9%** | **0.109%** | **0.157%** |
| Latino | 0.006% | 0.003% | 62.3% | 0.031% | 0.288% |
| South Asian | 0.007% | 0% | 31.1% | 0% | 0.013% |

Ala = alanine; Arg = arginine; Gly = glycine; Ile = isoleucine; Met = methionine; Thr = threonine; Val = valine.
